# Centre-level variation in the survival of patients receiving haemodialysis in India: findings from a nationwide private haemodialysis network

**DOI:** 10.1016/j.lansea.2024.100383

**Published:** 2024-03-13

**Authors:** Carinna Hockham, Arpita Ghosh, Ankit Agarwal, Kamal Shah, Mark Woodward, Vivekanand Jha

**Affiliations:** a The George Institute for Global Health, School of Public Health, Imperial College London, London, UK; b The George Institute for Global Health, UNSW International, New Delhi, India; c Prasanna School of Public Health, Manipal Academy of Higher Education, Manipal, India; d NephroPlus Dialysis Network, Hyderabad, India; e The George Institute for Global Health, UNSW, Sydney, Australia

**Keywords:** Haemodialysis, Dialysis centres, Mortality, Survival analysis, Frailty models

## Abstract

**Background:**

There are no large studies examining survival in patients receiving haemodialysis in India or considering centre-level effects on survival. We measured survival variation between dialysis centres across India and evaluated the extent to which differences are explained by measured centre characteristics.

**Methods:**

This is a multilevel analysis of patient survival in centres of the NephroPlus dialysis network consisting of 193 centres across India. Patients receiving haemodialysis at a centre for ≥90 days between April 2014 and June 2019 were included, with analyses restricted to centres with ≥10 such patients. The primary outcome was all-cause mortality, measured from 90 days after joining a centre. Proportional hazards models with shared frailty were used to model centre- and patient-level effects on survival.

**Findings:**

Amongst 23,601 patients (median age 53 years; 29% female), the unadjusted centre-specific 180-day Kaplan–Meier survival estimates ranged between 55% (95% confidence interval [CI] 38–80%) and 100%, with a median of 88% (interquartile interval 83%–92%). After accounting for multilevel factors, estimated 180-day survival ranged between 83% (73–89%) and 97% (95–98%), with 90% 180-day survival in the average centre. The mortality rate in patients attending rural centres was 32% (Hazard Ratio 1.32; 95% CI 1.06–1.65) higher than those at urban centres in adjusted analyses. Multiple patient characteristics were associated with mortality.

**Interpretation:**

This is the first national benchmark for survival amongst dialysis patients in India. Centre- and patient-level characteristics are associated with survival but there remains unexplained variation between centres. As India continues to widen dialysis access, ongoing quality improvement programs will be an important part of ensuring that patients experience the best possible outcomes at the point of care.

**Funding:**

This project received no external funding.

**Research in context**

**Evidence before this study**

Data on the clinical outcomes of patients receiving dialysis in India are limited. The few studies that are published are either single-centre, small, or over 10 years old. The largest study, conducted in Andhra Pradesh in South India, used claims data from a publicly funded insurance scheme between 2008 and 2012 to describe outcomes amongst 13,118 beneficiaries, and found that 10.2% of patients died within six months of starting haemodialysis. However, there was limited opportunity in the study to examine whether differences in survival existed between dialysis centres, as has been shown in other countries. As a result, major gaps exist in our understanding of dialysis outcome patterns in India, limiting efforts to improve care across the country. There is also, as yet, no national benchmark for survival in patients receiving dialysis. We aimed to address this knowledge gap using de-identified individual-level administrative data from the nationwide NephroPlus private dialysis network. Specifically, we measured the extent of variation in patient survival between 193 centres as well as the relative influence of centre- and patient-level characteristics on survival, using multilevel modelling.

**Added value of this study**

The NephroPlus database provides a unique opportunity to derive outcomes of patients receiving haemodialysis in India on a larger scale than previously possible—both in terms of patient numbers and geographical scope. After accounting for both centre- and patient-level factors across 193 centres in 20 states, we found that the estimated 180-day survival ranged between 83% (95% Confidence Interval [CI] 73–89%) and 97% (95% CI 95–98%) across centres, with a survival of 90% in the average centre. The mortality rate in patients registered at rural centres was 32% higher than those at urban centres, which is likely due in part to unmeasured differences in patient characteristics. Nevertheless, it highlights the unique challenges faced by rural centres. Numerous patient-level characteristics were associated with mortality, for example, catheter-based vascular access (higher mortality compared to access via an arteriovenous fistula or graft), and financial support for dialysis treatment through a government panel scheme or private insurance (lower mortality compared to out-of-pocket payment). The inclusion of centre-level factors reduced the variability in survival across dialysis centres by 31% when patient case mix was also accounted for. However, whether these factors, in particular rural *versus* urban centres, are directly related to mortality or are simply surrogate markers for other centre or patient characteristics is unclear.

**Implications of all the available evidence**

With ongoing efforts to broaden dialysis access in India, it is generally well-regarded that the number of patients receiving dialysis will increase. The variation in survival between centres observed in this study demonstrates that a collaborative quality improvement system in the country is needed, alongside overall improvements in healthcare infrastructure. Establishing national benchmarks for dialysis outcomes against which both other centres and changes over time can be compared is essential to drive this, and our findings represent a first step.

## Introduction

India has amongst the highest number of patients receiving chronic dialysis globally—estimated at around 175,000 individuals in 2018.^1^ Even so, only around one-third of patients who require kidney replacement therapy (KRT; dialysis or transplant) actually receive it.^1^ The number of patients on dialysis is increasing.^2^ This is due to a number of reasons, including the launch of the National Dialysis Service in 2016 to improve access to KRT,^1^^,^^3^ ongoing efforts to develop affordable dialysis systems,^4^ and the rising incidence of kidney failure in the country.^5^

Despite this growing burden, research into the clinical outcomes of dialysis patients in India is limited and there is currently no national benchmark for outcomes with this form of KRT.^3^^,^^6^ The few studies that are available are either single-centre,^7^, ^8^, ^9^, ^10^, ^11^, ^12^ have small sample sizes,^13^^,^^14^ or are over 10 years old.^15^, ^16^, ^17^ The largest study to date, involving 13,118 patients receiving haemodialysis through a publicly funded insurance scheme in Andhra Pradesh between 2008 and 2012, found that 13% of patients died within one year of starting treatment.^18^ Two percent had received a kidney transplant and almost half had stopped dialysis, presumed to have died. Whether these findings are generalisable to other states or dialysis providers is unknown, with 1-year mortality estimates from other states ranging between 10% and 29%.^13^^,^^16^

Alongside establishing national standards for dialysis outcomes in India, it is important to understand the multilevel effects of centre- and patient-level characteristics on these outcomes. It has been shown, in nephrology and other medical specialties, that clinical practice patterns and patient outcomes vary substantially between centres.^19^^,^^20^ For example, KRT registry data from the United Kingdom showed that the predicted 3-year survival of patients starting KRT in 46 centres in England varied between 60.2% and 78.7%, adjusting for age and sex.^21^ While differences in patient characteristics (or “case mix”) between centres may partially explain this variation, centre-level characteristics such as staffing, processes of care and patient volume also likely play a role.^19^ However, both the extent of between-centre variation and the relative influence of centre- and patient-level characteristics on dialysis outcomes are context-dependent, and observations made in Western countries will not be generalisable to India.

NephroPlus is the largest dialysis network in India.^3^ Across the network, information collected as part of routine care is entered into a central electronic medical record (eMR) database, capturing a wealth of longitudinal information on patients and their care. Until a national dialysis registry is established,^6^^,^^13^ this database provides a unique opportunity to derive patient outcomes on a larger scale than previously possible. Using this source, we aimed to: (i) establish a national benchmark for survival in patients receiving haemodialysis in India, (ii) quantify the variation in survival between centres, and (iii) examine centre- and patient-specific characteristics associated with survival.

## Methods

### Study setting

As at 2023, NephroPlus provides dialysis care to approximately 22,000 patients (∼10% of all those receiving dialysis) across 300 centres in 28 of the country's 36 states and union territories. During centre registration, patients consented to the use of their de-identified data for clinical research.

For the present study, we included all patients who registered with a NephroPlus centre in India between April 1, 2014 and June 30, 2019 and had received haemodialysis from the centre for more than 90 days. A minimum period of 90 days was chosen to avoid including patients with acute kidney injury receiving short-term dialysis, consistent with other registry-based studies on dialysis outcomes.^21^^,^^22^ Patients were followed until March 31, 2020 to allow a minimum of 6 months of follow-up for all patients and to avoid our findings being impacted by disruptions caused by COVID-19.

We followed the RECORD guidelines^23^ for reporting observational studies using routinely collected data.

### Outcome

The outcome was all-cause mortality, measured from 90 days after commencing dialysis at the NephroPlus centre until death, loss-to-follow-up or March 31, 2020, whichever occurred first. In the primary analysis, deaths were those that were explicitly recorded in the NephroPlus database. However, due to the potential for high loss to follow-up,^18^ and the high probability of rapid death for patients with kidney failure who stop receiving treatment, we conducted a sensitivity analysis in which we presumed that, in addition to the confirmed deaths, patients had likely died if they were no longer active in the database because they had been admitted to a hospital and not returned to the centre or had actively withdrawn from dialysis, and had not attended dialysis for more than 30 days prior to study end. Death was assumed to take place 30 days after a patient's last recorded dialysis session.

### Covariates

Centre characteristics included the year the centre opened, frequency of clinic attendance by a consultant nephrologist, and the number of beds, staff and active patients. Geographic factors were also considered, including region (North, South, East and West) and the tier of the city or town where the centre is located (Tiers I, II and III, corresponding to urban, semi-urban and rural areas, respectively, as defined by the Government of India).

Patient-level demographic, socioeconomic and lifestyle characteristics included age, sex, education, monthly household income (Indian Rupees), and smoking status. We also included payment method, which included out-of-pocket, government health scheme (e.g. the Central Government Health Scheme [CGHS] or Ex-Servicemen Contributory Health Scheme [ECHS]), and private insurance. Comorbidities were diabetes, coronary heart disease and heart failure, hypertension, hepatitis B and hepatitis C. Dialysis vintage prior to joining a NephroPlus centre was categorised as ≤30 days, >30 to ≤365 days and >365 days. The baseline number of dialysis sessions per week was calculated as the median number of days between consecutive session dates during the first 90 days after study entry, irrespective of subsequent changes in dialysis frequency. A median of 0–1.5 days was considered to indicate three sessions per week, a median of 2.0–3.0 days to indicate two sessions per week, and a median of 3.5–6.5 days to indicate one session per week. Vascular access at baseline was via arteriovenous fistula (AVF) or graft (AVG), permanent catheter, jugular catheter, or other. A fifth category for vascular access indicated patients who switched from a jugular catheter to an AVF/AVG between joining a centre and their data being extracted for the present study.

### Statistical analyses

#### Primary analyses

Proportional hazards models with shared frailty were used to model the hierarchical effects of centre-level characteristics on patient survival and account for patient clustering within centres.^24^ In the context of hazard models for time to event outcomes, the term ‘shared frailty’ denotes the exponential of the random effect, and its distribution was assumed to be log-normal. Centre-level and patient-characteristics were modelled separately and together. Hazard ratios with 95% confidence intervals (CI) were obtained, together with shared frailty estimates of the relative difference in log cumulative hazards between each centre and the “average” centre (the centre with a frailty closest to 1). These frailty estimates have a multiplicative effect on the baseline hazard function. Due to the very low rates of transplantation in this dataset (2.4% of patients), we did not treat transplantation as a competing risk of death.^3^

For each centre, we calculated the unadjusted Kaplan–Meier estimates of 180-day survival as the percentage of patients who were still alive 180 days after the start of follow-up, accounting for censoring. We also obtained model-predicted centre-specific values following adjustment for centre- and patient-level characteristics. The minimum and maximum values and interquartile interval (IQI) were reported for both the unadjusted and adjusted centre-specific estimates. For the latter, we also reported the predicted survival in the “average” centre. To estimate the adjusted centre-specific survival estimates, we first calculated estimates for a typical individual with covariates in the reference categories, conditional on the frailty term for a centre and using the baseline hazard estimates at each event time point. Conditional cumulative hazard and the conditional survival were then obtained using standard formulas.

To assess the extent to which between-centre variation was reduced after centre-level factors were added to the patient-level only model, we obtained the frailty variance from each model and calculated the percentage change. The frailty variance provides an overall measure of variation in survival across the centres and comparisons of this measure between the models provides an indication of how much of the variation is attributable to the modelled centre-level factors.

#### Sensitivity analyses

Sensitivity analyses were performed to examine the robustness of our findings. First, the frailty was assumed to be gamma distributed. Second, analyses were repeated with assumptions around the likelihood of death in patients who had stopped treatment. Third, we ran an imputation model under the missing at random (MAR) assumption. One hundred imputations were run using a fully conditional specification^25^^,^^26^ including the Nelson-Aalen estimate, event indicator and all variables included in primary analyses.^27^ Variables were imputed using the Multiple Imputation by Chained Equations (MICE) method of imputation. The individual-level binary variables (sex, smoking status, history of diabetes, history of heart disease or heart failure, history of hypertension, hepatitis B and hepatitis C) were imputed using logistic regression, and the categorical variables (education, monthly household income, dialysis frequency and vascular access) using polytomous logistic regression. We also imputed the centre-level variables frequency of nephrologist visits, number of beds, number of staff and number of patients using polytomous logistic regression. The hierarchical nature of data was not accounted for when imputed datasets were created.

Data cleaning and analyses were performed in R version 4.2.0. For analyses, the *survival*,^28^ *frailtyEM*,^29^ *coxme*,^30^ *mice*,^31^ *survminer*,^32^ *ggplot2*^33^ and *gtsummary* packages were used.^34^

### Ethical approval

Ethical approval for this study was waived by the Institutional Ethics Committee of The George Institute for Global Health India. This is due to our exclusive use of de-identified data from patients who had voluntarily consented to its use for clinical research at the time of registration at a NephroPlus centre, before any data was collected. A refusal to consent did not impact a patient's right to receive treatment, which was made clear to patients from the outset.

### Role of the funding source

Not applicable.

## Results

Of 58,047 patients registered across 300 NephroPlus centres between 2014 and 2019, 23,601 (41%) at 193 centres were eligible for inclusion and followed from 90 days after commencing maintenance haemodialysis at their treating centre until their death, loss to follow-up, or study end (March 31, 2020) (Supplementary Figure S1). Twenty-nine percent of patients were women and median baseline age was 53 (IQI 42–62) years (Table 1). Two-thirds of patients had received secondary school education, with 22% receiving tertiary education. The prevalence of diabetes and hypertension was high (37% and 75%, respectively). Over 90% of centres had access to a nephrologist and 55% had a nephrologist who attended clinic at least once a week (Table 2). Median follow-up time was 316 (IQI 148–654) days.

**Table 1:** Baseline patient characteristics, reported by sex.

Characteristics	Female	Male	Overall
	N = 6879	N = 16,713	N = 23,601
**Sex**			n = 23,592 (∼100)
Female	–	–	6879 (29)
**Age (years) at 1**st **dialysis session at NephroPlus, n (%)**			
**Median (IQI)**	53 (42–62)	53 (42–62)	53 (42–62)
<18	75 (1)	195 (1)	270 (1)
18–29	480 (7)	1168 (7)	1648 (7)
30–44	1476 (22)	3644 (22)	5122 (22)
45–59	2686 (39)	6484 (39)	9175 (39)
60–69	1571 (23)	3633 (22)	5205 (22)
≥70	591 (9)	1589 (10)	2181 (9)
**Time on dialysis before coming to NephroPlus, n (%)**			
≤30 days	3968 (58)	9585 (57)	13,559 (58)
>30 & ≤365 days	1474 (21)	3829 (23)	5305 (23)
>365 days	1437 (21)	3299 (20)	4737 (20)
**Education, n (%)**	4858 (71)	12,068 (72)	16,929 (72)
No schooling	1364 (28)	2323 (19)	3688 (22)
1st–5th grade	559 (12)	1165 (10)	1725 (10)
6th–12th grade	2139 (44)	5671 (47)	7811 (46)
Beyond 12th grade	796 (16)	2909 (24)	3705 (22)
**Monthly household income, n (%)**	n = 4362 (63)	n = 11,152 (67)	n = 15,517 (66)
<5000 INR	265 (6)	705 (6)	970 (6)
5000–15,000 INR	2980 (68)	7610 (68)	10,593 (68)
16,000–50,000 INR	994 (23)	2572 (23)	3566 (23)
>50,000 INR	123 (3)	265 (2)	388 (3)
**Payment, n (%)**			
Cash	3284 (48)	7115 (43)	10,403 (44)
Panel (Government subsidy)	3251 (47)	8907 (53)	12,161 (52)
Private insurance	344 (5)	691 (4)	1037 (4)
**Vascular access, n (%)**	n = 4399 (64)	n = 11,141 (67)	n = 15,542 (66)
Arteriovenous fistula or graft	2477 (56)	6768 (61)	9245 (60)
Permanent catheter	218 (5)	345 (3)	564 (4)
Jugular catheter to AVF/AVG	867 (20)	2108 (19)	2976 (19)
Jugular catheter	683 (16)	1585 (14)	2268 (15)
Other	154 (4)	335 (3)	489 (3)
**Dialysis frequency, n (%)**	n = 6798 (99)	n = 16,557 (99)	n = 23,362 (99)
Thrice a week	1397 (21)	3501 (21)	4898 (21)
Twice a week	4597 (68)	11,149 (67)	15,751 (67)
Once a week or less frequently	804 (12)	1907 (12)	2713 (12)
**Smoking status, n (%)**	n = 6865 (99.8)	n = 16,699 (99.9)	n = 23,573 (99.9)
Smoker	99 (1)	1777 (11)	1878 (8)
**History of heart attack or heart failure, n (%)**	n = 6831 (99)	n = 16,623 (99.5)	n = 23,463 (99)
Yes	227 (3)	728 (4)	955 (4)
**History of diabetes, n (%)**	n = 6867 (99.8)	n = 16,699 (99.9)	n = 23,575 (99.9)
Yes	2323 (34)	6323 (38)	8647 (37)
**History of hypertension, n (%)**	n = 6867 (99.8)	n = 16,698 (99.9)	n = 23,574 (99.9)
Yes	5082 (74)	12,512 (75)	17,601 (75)
**Hepatitis C, n (%)**	n = 6490 (94)	n = 15,981 (96)	n = 22,479 (95)
Yes	521 (8)	1270 (8)	1791 (8)
**Hepatitis B, n (%)**	n = 6489 (94)	n = 15,979 (96)	n = 22,476 (95)
Yes	129 (2)	473 (3)	602 (3)

For percentages, the denominator is the number of individuals without a missing value.

**Table 2:** Centre characteristics, with corresponding patient numbers and unadjusted mortality rate.

	Centres	Patients	Deaths	Unadjusted mortality per 100 patient-years	P-value
	N (%^a^)	N (%^b^)	N (%^b^)		
**Centre type**	n = 193				<0.01
Captive	183 (95)	22,173 (94)	6282 (28)	23.5	
Stand-alone	10 (5)	1428 (6)	355 (25)	16.3	
**Type of city**	n = 193				<0.01
Tier I (urban)	52 (27)	5072 (23)	1152 (23)	18.0	
Tier II (semi-urban)	76 (39)	10,951 (46)	2850 (26)	21.4	
Tier III (rural)	65 (34)	7578 (32)	2635 (35)	28.8	
**Region**	n = 193				<0.01
North	49 (25)	6608 (28)	1553 (24)	20.3	
South	83 (43)	11,070 (47)	3609 (33)	26.0	
East	32 (17)	2949 (13)	796 (27)	24.7	
West	29 (15)	2974 (13)	679 (23)	16.4	
**Year centre started**	n = 193				<0.01
Before 2015	28 (15)	4567 (19)	1297 (28)	19.6	
2015	33 (17)	5782 (25)	1555 (27)	20.1	
2016	33 (17)	4402 (19)	1410 (32)	24.8	
2017	40 (21)	5637 (24)	1875 (33)	28.5	
2018	45 (23)	2681 (11)	426 (16)	21.6	
2019	14 (7)	532 (2)	74 (14)	23.8	
**Availability of in-house nephrologist**	n = 191	n = 23,553			0.3
No	13 (7.0)	673 (3)	118 (18)	22.1	
Yes	178 (93)	22,880 (97)	6516 (29)	23.0	
**Nephrologist visit frequency**	n = 191	n = 23,553			<0.01
No coverage	12 (6)	620 (3)	117 (19)	23.1	
1-2 times/month	75 (39)	8551 (36)	2935 (34)	29.0	
1-3 times/week	34 (18)	2378 (10)	575 (24)	21.6	
Daily	70 (37)	12,004 (51)	3007 (25)	19.3	
**Availability of duty doctor**	n = 191	n = 23,553			<0.01
No coverage	85 (45)	7132 (30)	1907 (27)	25.2	
Visiting	40 (21)	4883 (21)	1171 (24)	18.8	
Full time	66 (35)	11,538 (49)	3556 (31)	23.5	
**Number of beds**	n = 191	n = 23,553			<0.01
1–5	29 (15)	1469 (6)	386 (26)	28.8	
6–11	82 (43)	6581 (28)	1742 (27)	23.3	
12–105	80 (42)	15,503 (66)	4506 (29)	22.5	
**Number of staff**	n = 188	n = 23,327			<0.01
1–4	37 (20)	2357 (10)	631 (27)	25.8	
5–8	67 (36)	5217 (22)	1516 (29)	26.2	
9–67	84 (45)	15,753 (68)	4417 (28)	21.6	
**Number of patients**	n = 191	n = 23,553			<0.01
2–28	47 (25)	2658 (11)	691 (26)	26.7	
29–66	71 (37)	6651 (28)	1752 (26)	22.8	
67–675	73 (38)	14,244 (61)	4191 (29)	22.5	

a Denominator is number of centres.

b Denominator is number of individuals.

There were no discernible differences in the demographic and socioeconomic characteristics between patients who were and were not included in the final cohort (Supplementary Table S1).

### Variation in survival between centres

**Fig. 1:**
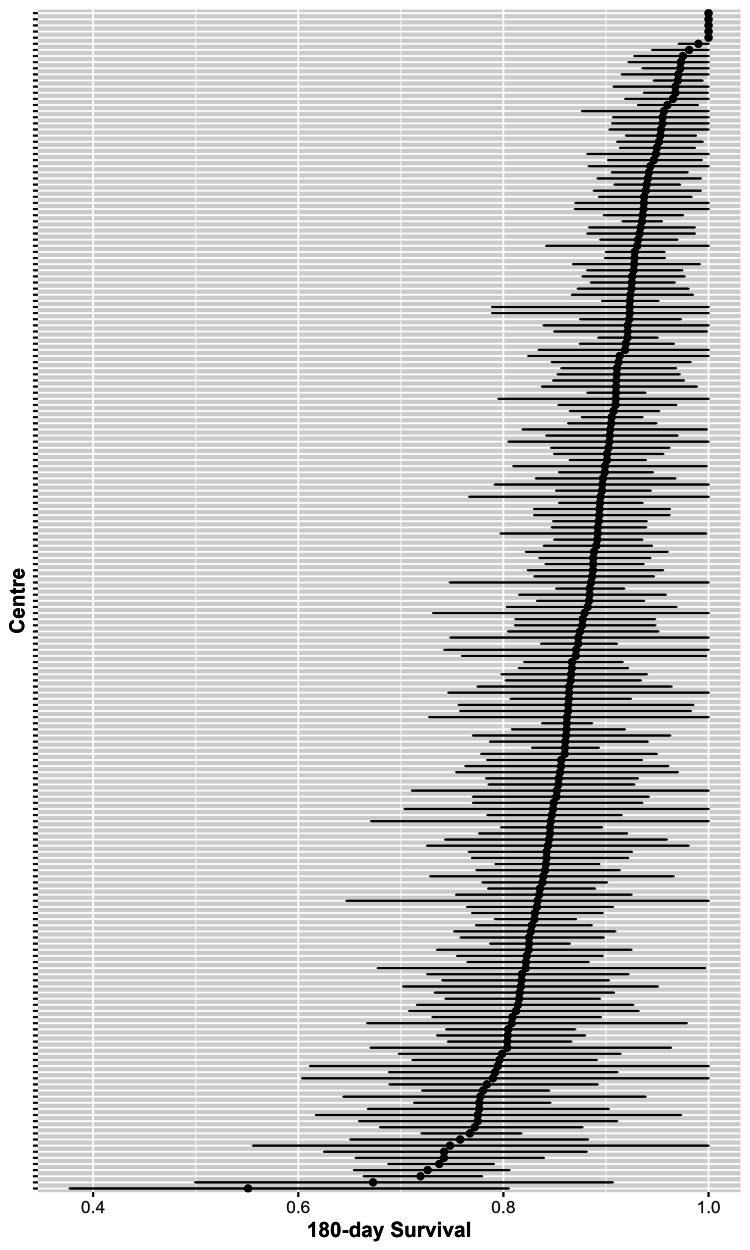
Caterpillar plot showing the unadjusted 180-day survival in each of the 192 centres included in this study.

**Fig. 2:**
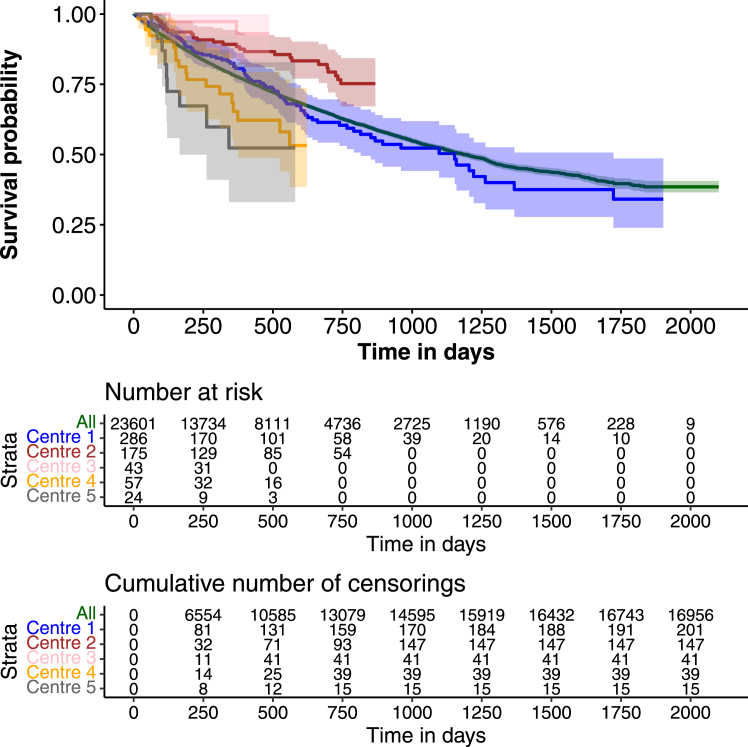
Kaplan–Meier plot of survival, overall and by centre. For aesthetic purposes, survival curves for only five centres are shown. One centre was randomly selected within each of the five equal fifths of the mortality rate.

Overall, 6637 (28%) patients receiving haemodialysis died over a median follow-up of 10 months. 16,864 (71%) patients were alive 180 days after study entry, 2615 (11%) had died and 4150 (18%) were lost to follow-up. At the centre level, the unadjusted 180-day Kaplan–Meier survival estimates ranged between 55% (95% CI 38–80%) and 100%, with a median of 88% (IQI 83%–92%) (Figs. 1 and 2). After accounting for differences in centre- and patient-level factors, the estimated 180-day survival at the average centre was 90% (95% CI 85–94%) and ranged between 83% (95% CI 73–89%) and 97% (95% CI 95–98%). The IQI for the adjusted 180-day survival was 89% and 92%.

### Associations between and centre- and patient-level factors with survival

#### Centre-only model

Adjusting for centre-level characteristics only, mortality was lower amongst patients at centres in the Western zone compared to those in the Northern zone (HR 0.78 [95% CI 0.63–0.96]) (Supplementary Figure S2). No other centre factors were associated with mortality.

#### Patient-only model

When only patient case mix was adjusted for, mortality was 15–20% lower in adults aged 30–44 and 45–59 years compared to those under 30 (HR 0.79 [0.72–0.87] and 0.85 [0.77–0.93], respectively) (Supplementary Figure S3). Those with a secondary school education and above had a lower mortality compared to patients with no formal education (HR 0.77 [0.71–0.83] for those who reached grade 12 and HR 0.67 [0.61–0.74] for those who surpassed grade 12). This was also true for patients whose monthly household income was INR 50,000 or more (HR 0.71 [0.56–0.92], compared to those with a monthly household income of less than INR 5000). A lower mortality was also observed for patients who did not pay for their treatment out-of-pocket, with an HR of 0.72 (95% CI 0.66–0.78) for government-subsidised patients and an HR of 0.75 (95% CI 0.64–0.88) for privately insured patients. There was an inverse relationship between mortality and dialysis vintage, with those receiving dialysis for at least a year prior to joining a centre having a 17% lower rate of mortality than those who started dialysis less than 30 days before joining (HR 0.83 [0.77–0.89]).

Presence of diabetes (HR 1.35 [95% 1.28–1.43]) was associated with a higher mortality rate (Supplementary Figure S3). In terms of dialysis practices, compared to patients with an AV fistula or graft at baseline, mortality was almost double in patients with a temporary jugular catheter (HR 1.96 [1.80–2.14]), around 75% higher in those with a tunnelled catheter or other access type (HR 1.73 [1.49–2.01] and 1.78 [1.51–2.09], respectively), and 18% higher in those who started dialysis on a jugular catheter and later switched to an AV fistula or graft (HR 1.18 [1.09–1.28]). Further, the receipt of dialysis fewer than three times per week was associated with an 8% mortality increase, albeit with a 95% CI close to spanning 1 (HR 1.08 [1.01–1.15]).

#### Final model with centre- and patient-level characteristics

**Fig. 3:**
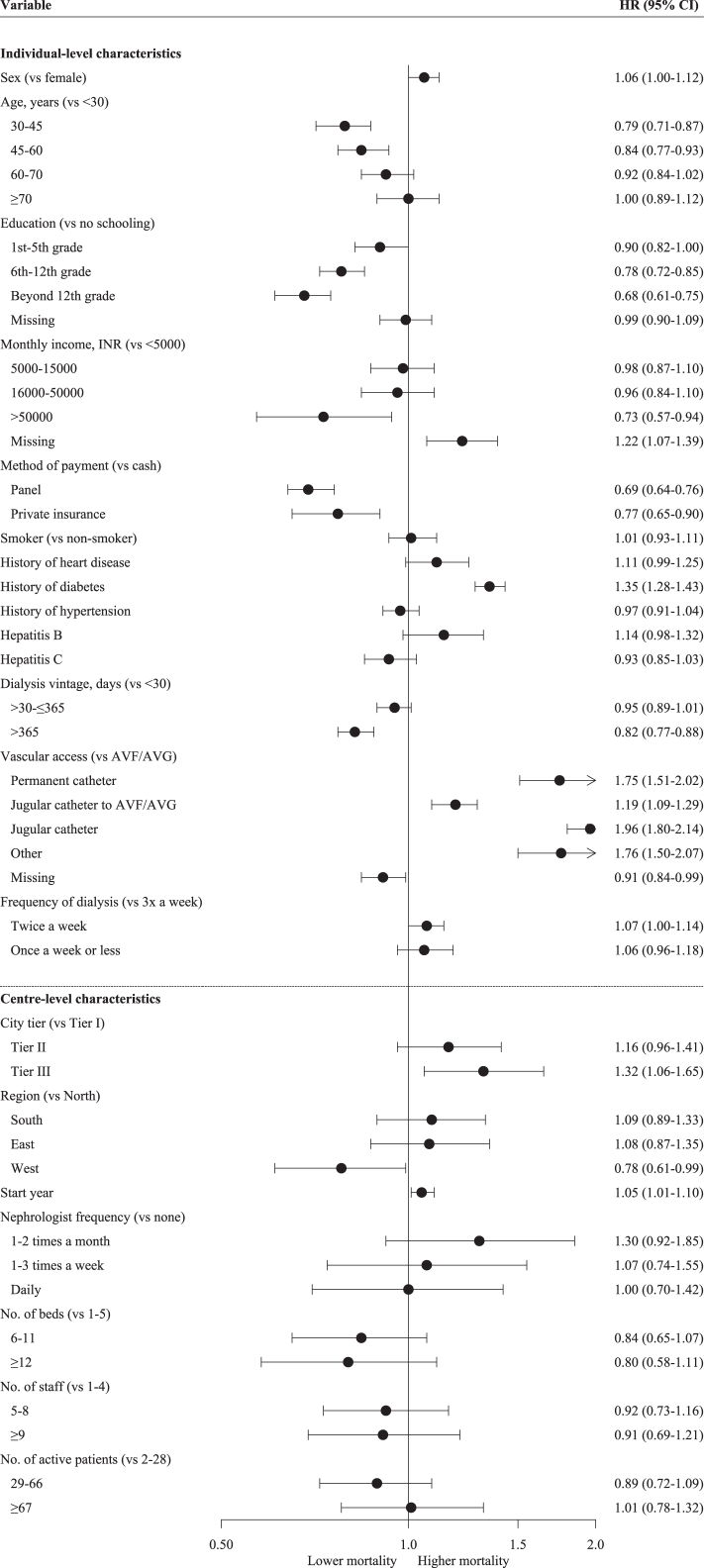
Multilevel adjusted associations between patient- and centre-level characteristics and all-cause mortality.

When patient case mix was also taken into account, mortality was found to be higher among patients treated in rural centres compared to those in urban areas (HR 1.32 [1.06–1.65]) (Fig. 3). The association between region and mortality was unchanged. All associations between patient characteristics and mortality were similar between the patient-only model and the final model (Supplementary Figure S3 and Fig. 3).

The addition of centre-level characteristics to the patient-only model reduced the variation in survival across dialysis centres (the frailty variance) by 31%. The centre factors that were included in the analyses therefore accounted for 31% of the observed variation in survival when patient case mix was also accounted for, whilst 69% of the variation remained unexplained.

### Sensitivity analyses

In the sensitivity analyses including both confirmed and presumed deaths, the adjusted 180-day survival at the average centre was 90% (95% CI 85–94%) and ranged between 83% (95% CI 74–89%) and 97% (95% CI 95–98%). For all other sensitivity analyses, our findings for risk factor associations were largely unchanged (Supplementary Table S2).

## Discussion

This is the largest study—in size and geographic scope—of dialysis outcomes in India. In a cohort of 23,601 patients receiving dialysis treatment, we found that, 71% of patients were alive after 6 months. Between centres, 180-day survival ranged from 55% to 100%—narrowing to between 83% and 97% after accounting for centre and patient characteristics. Thirty-one percent of the variation in survival was attributable to the centre factors included in our final model, which themselves may be surrogate markers of other centre or patient characteristics. There was also considerable variation (69%) that was not explained by our model, suggesting that unmeasured characteristics (centre or patient) are at play.

The observed 180-day mortality is higher than the 10.2% for the equivalent time reported in a study from Andhra Pradesh, which used insurance claims data to examine dialysis outcomes.^18^ This may, in part, be explained by differences in reporting between the two databases, with the collection of death information being less systematic in the earlier study. Furthermore, a high percentage of NephroPlus patients paid for their treatment out of pocket—in contrast to the study from Andhra Pradesh, which only included beneficiaries of a publicly funded health insurance scheme. Out-of-pocket expenditure is most often borne by those who are least able to afford it and can have catastrophic economic and health consequences.^35^ Indeed, we found that mortality was highest in those who paid for their treatment out of pocket than those whose treatment was government-subsidised or covered by private insurance. It is also worth noting that our cohort had a higher percentage of patients over the age of 60 years (31% *versus* 15% in Shaikh et al.), another known risk factor for mortality amongst patients with kidney failure.^36^^,^^37^ The observed heterogeneity between these two studies is therefore not surprising, but does demonstrate the need for more widespread data collection on dialysis outcomes in India.

Comparisons with findings from elsewhere reveal the stark global inequities in dialysis outcomes.^38^ For instance, the 29% known mortality observed in this study over a median follow-up of 10 months is double the 14% of patients who died over a median follow-up of 16 months in phase 4 (2009–2011) of the Dialysis Outcomes and Practice Patterns Study (DOPPS), a retrospective cohort study of incident dialysis patients living mostly in high-income Western countries and Japan.^39^ It is also higher than the 16% mortality observed over a median follow-up of 17 months in the Gulf Cooperation Council (GCC) DOPPS phases 5 and 6 (2012–2018).^40^ In sub-Saharan Africa, a 2021 review (25 studies, 4228 participants) reported a pooled mortality of 31% over a mean duration of 4 years.^41^ However, mortality risk varied considerably between studies and countries, and was higher in incident patients, who accounted for 24% of the total pooled population.^41^ In contrast, 58% of patients in our cohort had spent 30 days or less on dialysis prior to joining NephroPlus, which might partly explain the higher mortality rate in our study. Whilst comparison with the pooled estimate from sub-Saharan Africa is made difficult by the low-to-medium quality of the studies included in the review, our findings support previous assertions that kidney failure deaths in India are higher than expected given its sociodemographic index.^1^^,^^42^

To drive improvement, standardised, prospective data collection on dialysis outcomes is needed: to enhance accountability and identify shortfalls in dialysis care. Efforts are underway to establish such a system in India,^3^ but this will take time given various challenges.^13^ In the meantime, dialysis networks such as NephroPlus, with their integrated electronic data capture systems, provide a unique opportunity to evaluate dialysis outcomes across most of India and establish a national benchmark against which to compare the outcomes of other centres as well as changes over time. We found that the adjusted estimated centre-specific 180-day survival ranged between 83% and 97%, with a survival of 90% in the average centre. As has been shown in other settings,^19^^,^^43^ comparisons between centres can stimulate quality improvement and encourage transparency between centres to allow knowledge sharing. Whether it is feasible to conduct annual comparisons—like those performed by registries elsewhere^44^—warrants further attention. Importantly though, such comparisons would need to be made in a collaborative environment with an openness to share best practices so that low-performing centres, or those operating in more challenging environments, can learn from high-performing ones.

Given the variation in survival between centres, a key objective was to understand the factors that are driving this variation. In the study from Andhra Pradesh, worse mortality was reported for patients receiving treatment at public *versus* private dialysis centres (HR 1.07 [1.03–1.12]).^18^ However, the authors were unable to account for differences in case mix between centres, limiting interpretation. As yet, no study has compared the mortality rate in centres operating in different tiers within India, although comparisons at the patient level have shown stark disparities.^1^ In the present study, rural centres were associated with a 32% higher mortality than urban centres, adjusting for measured patient factors (HR 1.32 [1.06–1.65]). This adjusted HR differs considerably from the unadjusted estimate (1.20 [0.99–1.46]), suggesting that unmeasured patient characteristics are likely to be confounding this association. Indeed, data from India and elsewhere suggest that rural populations have a higher risk of experiencing worse health outcomes, making the possibility of residual confounding strong.^19^^,^^45^ Due to the routinely collected nature of the NephroPlus dataset, the only socioeconomic factors available for analysis were education and monthly household income. Other patient-level factors that would be of particular interest include travel distance to a centre, employment status and occupation, access to critical medications such as erythropoiesis-stimulating agents (ESAs), anti-hypertensives and those for cardiovascular risk management, vascular access care, and nutritional status or food security. Whether rural centres are struggling to deliver evidence-based KRT with current resources or are simply a surrogate marker for a higher-risk patient population independent of kidney impairment remains unclear. Nevertheless, the poor mortality outcomes observed in this study, and in Andhra Pradesh, suggests that government subsidy beyond the direct costs of dialysis and erythropoietin administration is urgently needed, especially for rural populations. This includes additional support for the prevention and long-term management of major complications associated with dialysis, including cardiovascular disease and mineral bone disease.

In contrast to previous research,^46^^,^^47^ we did not find evidence for an association between the number of beds at a centre and mortality nor between the frequency of nephrologist visits and mortality. The lack of an association may simply be due to the small percentage (6%) of centres with no nephrologist coverage, or it might be that centres with better nephrologist coverage possess other features that more strongly predict mortality; for example, it is possible that nephrologist-led centres are referred patients with more complex or severe disease.

Patient characteristics that were associated with higher mortality were presence of diabetes and any vascular access that is not an AV fistula or graft. Those associated with lower mortality included younger age, higher socioeconomic status (based on education or monthly household income) and longer dialysis vintage. Not having to pay for dialysis treatment out of pocket was also strongly associated with lower mortality. All patient-level effects were largely unchanged by centre factor adjustment and, for the most part, are consistent with existing knowledge. The lack of a clear association between the frequency of dialysis sessions and mortality is worth noting, given the globally accepted standard of thrice-a-week delivery. Recent studies have suggested that incremental dialysis may be associated with equivalent outcomes, at least in the early years of dialysis.^48^ In India, twice-a-week haemodialysis is the most common practice.^1^

A major strength of this study is the use of individual-level data to adjust for centre differences in case mix, as opposed to aggregates of patient-level data. However, it is important to consider that limited individual-level data on dialysis patients in India makes it difficult to assess the representativeness of our cohort. At a centre level, the low percentage (5%) of standalone centres, combined with high percentage (93%) of centres that have access to an in-house nephrologist, might not be representative of other dialysis providers in the country. Nevertheless, the extensive coverage of the NephroPlus network, together with the mix of urban, peri-urban and rural centres represented, makes this the most comprehensive dataset of dialysis patients and their outcomes in India to date. There are also several limitations that relate to the quality and availability of data in a dataset primarily collected for administrative purposes. First, we were unable to assess other clinical outcomes, such as the development or progression of comorbidities, due to these not being uniformly documented over time. Cardiovascular disease and infections are responsible for two-thirds of overall mortality.^1^ Being able to distinguish between these causes of death could have important implications for the *types* of interventions that are needed to improve outcomes. Measures around quality of life, management of complications and process indicators (e.g. small solute clearance) were also not available. There were also several patient-level characteristics that could not be explored, as these were not captured in the database. Unmeasured characteristics of particular interest have been described above but it would also be useful to have information on biochemical parameters so that centre-level summaries of measures such as haemoglobin, phosphate and serum albumin could be incorporated into the multilevel models.^19^ Furthermore, we were only able to examine the association between the direct costs of dialysis with mortality, whilst indirect costs are also likely to be important. It is also important to bear in mind that there are more subtle influences, such as the ethos and culture of the organisation,^19^ that may contribute to patient outcomes, but which are difficult to measure quantitatively. Finally, whilst NephroPlus has broad geographic coverage, it is just one provider in India and findings here may not be generalisable to outcomes observed in public hospitals or to patients receiving other forms of dialysis.

This is the largest study to date to investigate survival outcomes among patients receiving haemodialysis in India, and the first to compare survival between dialysis centres in multilevel analyses. We demonstrate variation in patient survival between dialysis centres, with both centre- and patient-level characteristics associated with survival. Based on the available data, patient survival is worse among patients attending rural centres, but this likely reflects the complex needs of the communities that these centres serve. As access to dialysis is expanded across India, administrative databases from providers like NephroPlus will be an invaluable resource with which to monitor dialysis outcomes, between centres and over time.

## Contributors

AG and VJ contributed to study concept and design. AA and KS contributed to acquisition of data. AG performed all statistical analyses and had full access to the data. CH, AG, KS, MW and VJ contributed to interpretation of the data. CH and AG did the literature search. CH wrote the first draft of the manuscript. AG, AA, KS, MW and VJ critically appraised the manuscript for important intellectual content. MW and VJ supervised the study. AG, AA and KS had full direct access to and verified the underlying data. AG takes responsibility for the integrity of the data and the accuracy of the data analysis. All authors accept responsibility to submit for publication.

## Data sharing statement

Data held within the NephroPlus database were shared for this project as part of a collaboration between academic researchers and NephroPlus. NephroPlus is open to data access requests from bona fide researchers, which will be considered on a case-by-case basis. All R programming code is available upon reasonable request.

## Declaration of interests

AG and CH have no conflicts of interest to declare. AA is employed by NephroPlus. KS is the Co-Founder and Director of NephroPlus. MW has recently been a consultant to Amgen and Freeline. VJ has received grant funding from GSK, Baxter Healthcare, and Biocon and honoraria from Bayer, AstraZeneca, Boehringer Ingelheim, NephroPlus and Zydus Cadilla, under the policy of all honoraria being paid to the organization.
