# Supplementary material for: Centre-level variation in the survival of patients receiving haemodialysis in India: findings from a nationwide private haemodialysis network
Source: Lancet Reg Health Southeast Asia. 2024 Mar 13;23:100383. doi: 10.1016/j.lansea.2024.100383 (PMC11004392; doi:10.1016/j.lansea.2024.100383)
Supplement: Supplementary Tables S1 and S2 and Supplementary Figures S1–S3 [file mmc1.docx]

# Supplementary Table & Figure legends

**Supplementary Table 1** Demographic and socioeconomic characteristics of patients who were not included in the final study cohort and those who were included.

**Supplementary Table 2** Results from sensitivity analyses.

**Supplementary Figure 1** Participant flow chart.

**Supplementary Figure 2** Adjusted analyses with only centre-level characteristics included.

**Supplementary Figure 3** Adjusted analyses with only patient-level characteristics included.

**Supplementary Table 1** Demographic and socioeconomic characteristics of patients who were not included in the final study cohort and those who were included. Numbers depict N (%).

| **Variables** | **Not included (N = 34,429)** | **Included (N=23,601)** |
| --- | --- | --- |
| **Sex** |  |  |
| Female | 9968 (29%) | 6879 (29%) |
| Male | 24455 (71%) | 16713 (71%) |
| Missing | 6 | 9 |
| **Age (years)** |  |  |
| <18 | 735 (2.3%) | 270 (1.1%) |
| 18-29 | 2267 (7.1%) | 1648 (7.0%) |
| 30-44 | 6636 (21%) | 5122 (22%) |
| 45-59 | 11891 (37%) | 9175 (39%) |
| 60-69 | 7182 (22%) | 5205 (22%) |
| ≥70 | 3217 (10%) | 2181 (9.2%) |
| Missing | 2501 | 0 |
| **Education** |  |  |
| No schooling | 4129 (28%) | 3688 (22%) |
| 1^st^-5^th^ grade | 1301 (8.7%) | 1725 (10%) |
| 6^th^-12^th^ grade | 6519 (44%) | 7811 (46%) |
| Beyond 12^th^ grade | 2973 (20%) | 3705 (22%) |
| Missing | 19507 | 6672 |
| **Monthly household income (INR**) |  |  |
| 5000-15000 | 385 (2.4%) | 970 (6.3%) |
| 16000-50000 | 12792 (80%) | 10593 (68%) |
| >50000 | 2430 (15%) | 3566 (23%) |
| Missing | 309 (1.9%) | 388 (2.5%) |
| 5000-15000 | 18513 | 8084 |
| **Type of city** |  |  |
| Tier I (urban) | 8965 (26%) | 5072 (21%) |
| Tier II (semi-urban) | 15701 (46%) | 10951 (46%) |
| Tier III (rural) | 9339 (27%) | 7578 (32%) |
| Missing | 424 | 0 |
| **Region** |  |  |
| North | 9983 (29%) | 6608 (28%) |
| South | 14792 (43%) | 11070 (47%) |
| East | 4578 (13%) | 2949 (12%) |
| West | 5076 (15%) | 2974 (13%) |

**Supplementary Table 2** Results from sensitivity analyses

| **Variables** | **Hazard Ratio (95% Confidence Interval)** | | | | |
| --- | --- | --- | --- | --- | --- |
|  | **Primary analysis (log-normal frailty)** | **Gamma frailty** | **Including presumed deaths** | **Complete case analysis** | **Multiple imputation** |
| **Individual-level characteristics** | | | | | |
| **Sex (vs female)** | 1.06 (1.00-1.12) | 1.06 (1.00-1.13) | 1.06 (1.01-1.12) | 1.04 (0.96-1.13) | 1.07 (1.01-1.13) |
| Age-years (vs <30) |  |  |  |  |  |
| 30-45 | 0.79 (0.71-0.87) | 0.79 (0.71-0.87) | 0.79 (0.72-0.87) | 0.73 (0.64-0.84) | 0.78 (0.71-0.86) |
| 45-60 | 0.84 (0.77-0.93) | 0.84 (0.77-0.93) | 0.84 (0.77-0.91) | 0.81 (0.71-0.91) | 0.83 (0.76-0.91) |
| 60-70 | 0.92 (0.84-1.02) | 0.92 (0.84-1.02) | 0.90 (0.82-0.99) | 0.88 (0.77-1.00) | 0.91 (0.83-1.00) |
| ≥70 | 1.00 (0.89-1.12) | 1.00 (0.89-1.12) | 0.99 (0.89-1.11) | 0.95 (0.81-1.11) | 0.99 (0.88-1.10) |
| **Education (vs no schooling)** |  |  |  |  |  |
| 1^st^-5^th^ grade | 0.90 (0.82-1.00) | 0.90 (0.82-1.00) | 0.89 (0.81-0.99) | 0.91 (0.81-1.02) | 0.91 (0.83-1.00) |
| 6^th^-12^th^ grade | 0.78 (0.72-0.85) | 0.78 (0.72-0.85) | 0.78 (0.72-0.84) | 0.78 (0.71-0.86) | 0.78 (0.72-0.84) |
| Beyond 12^th^ grade | 0.68 (0.61-0.75) | 0.68 (0.61-0.75) | 0.67 (0.60-0.73) | 0.68 (0.60-0.76) | 0.66 (0.59-0.72) |
| Missing | 0.99 (0.90-1.09) | 1.00 (0.91-1.10) | 0.97 (0.89-1.07) |  |  |
| **Monthly income, INR (vs <5000)** |  |  |  |  |  |
| 5000-15000 | 0.98 (0.87-1.10) | 0.98 (0.87-1.10) | 1.01 (0.90-1.13) | 0.95 (0.85-1.08) | 0.95 (0.85-1.06) |
| 16000-50000 | 0.96 (0.84-1.10) | 0.96 (0.84-1.10) | 1.00 (0.88-1.14) | 0.96 (0.83-1.11) | 1.08 (0.94-1.23) |
| >50000 | 0.73 (0.57-0.94) | 0.73 (0.57-0.94) | 0.76 (0.60-0.98) | 0.77 (0.59-1.00) | 0.93 (0.74-1.18) |
| Missing | 1.22 (1.07-1.39) | 1.23 (1.08-1.40) | 1.28 (1.12-1.45) |  |  |
| **Method of payment (vs cash)** |  |  |  |  |  |
| Panel | 0.69 (0.64-0.76) | 0.69 (0.63-0.76) | 0.70 (0.64-0.77) | 0.72 (0.64-0.82) | 0.71 (0.65-0.77) |
| Private insurance | 0.77 (0.65-0.90) | 0.77 (0.65-0.90) | 0.73 (0.62-0.85) | 0.86 (0.70-1.06) | 0.75 (0.65-0.88) |
| **Smoker (vs non-smoker)** | 1.01 (0.93-1.11) | 1.01 (0.93-1.11) | 1.02 (0.93-1.11) | 0.98 (0.87-1.09) | 1.00 (0.92-1.09) |
| **History of heart disease** | 1.11 (0.99-1.25) | 1.11 (0.99-1.26) | 1.10 (0.98-1.23) | 1.14 (0.97-1.34) | 1.13 (1.00-1.27) |
| **History of diabetes** | 1.35 (1.28-1.43) | 1.36 (1.29-1.43) | 1.34 (1.28-1.42) | 1.39 (1.29-1.49) | 1.33 (1.26-1.40) |
| **History of hypertension** | 0.97 (0.91-1.04) | 0.97 (0.91-1.04) | 0.96 (0.90-1.03) | 1.00 (0.91-1.10) | 0.95 (0.89-1.01) |
| **Hepatitis B** | 1.14 (0.98-1.32) | 1.14 (0.98-1.32) | 1.17 (1.01-1.34) | 1.22 (0.99-1.49) | 1.15 (0.99-1.33) |
| **Hepatitis C** | 0.93 (0.85-1.03) | 0.94 (0.85-1.03) | 0.93 (0.85-1.02) | 1.01 (0.90-1.14) | 0.95 (0.87-1.04) |
| **Dialysis vintage, days (vs <30)** |  |  |  |  |  |
| >30-≤365 | 0.95 (0.89-1.01) | 0.95 (0.89-1.01) | 0.96 (0.91-1.03) | 0.95 (0.87-1.03) | 0.99 (0.93-1.06) |
| >365 | 0.82 (0.77-0.88) | 0.82 (0.77-0.88) | 0.82 (0.76-0.88) | 0.83 (0.75-0.91) | 0.88 (0.82-0.95) |
| **Vascular access (vs AVF/AVG)** |  |  |  |  |  |
| Permanent catheter | 1.75 (1.51-2.02) | 1.75 (1.51-2.03) | 1.79 (1.55-2.07) | 1.89 (1.58-2.26) | 1.64 (1.42-1.89) |
| Jugular catheter to AVF/AVG | 1.19 (1.09-1.29) | 1.19 (1.10-1.29) | 1.20 (1.11-1.30) | 1.28 (1.16-1.42) | 1.20 (1.11-1.30) |
| Jugular catheter | 1.96 (1.80-2.14) | 1.96 (1.80-2.14) | 2.04 (1.87-2.21) | 2.33 (2.09-2.58) | 1.92 (1.77-2.09) |
| Other | 1.76 (1.50-2.07) | 1.77 (1.51-2.08) | 1.91 (1.64-2.22) | 1.94 (1.60-2.36) | 1.68 (1.44-1.96) |
| Missing | 0.91 (0.84-0.99) | 0.91 (0.84-0.99) | 0.96 (0.89-1.04) |  |  |
| **Frequency of dialysis (vs 3x a week)** |  |  |  |  |  |
| Twice a week | 1.07 (1.00-1.14) | 1.07 (1.00-1.14) | 1.07 (1.00-1.15) | 1.07 (0.97-1.17) | 1.07 (1.00-1.15) |
| Once a week or less | 1.06 (0.96-1.18) | 1.06 (0.96-1.17) | 1.11 (1.01-1.23) | 1.03 (0.89-1.19) | 1.04 (0.94-1.16) |
| **Centre-level characteristics** | | | | | |
| **City tier (vs Tier I)** |  |  |  |  |  |
| Tier II | 1.16 (0.96-1.41) | 1.20 (0.98-1.48) | 1.16 (0.96-1.40) | 1.17 (0.93-1.47) | 1.14 (0.95-1.38) |
| Tier III | 1.32 (1.06-1.65) | 1.33 (1.05-1.69) | 1.32 (1.06-1.64) | 1.35 (1.03-1.77) | 1.31 (1.06-1.63) |
| **Region (vs North)** |  |  |  |  |  |
| South | 1.09 (0.89-1.33) | 1.10 (0.88-1.36) | 1.09 (0.90-1.33) | 1.08 (0.87-1.35) | 1.12 (0.91-1.37) |
| East | 1.08 (0.87-1.35) | 1.09 (0.86-1.38) | 1.07 (0.87-1.32) | 1.05 (0.81-1.34) | 1.08 (0.87-1.34) |
| West | 0.78 (0.61-0.99) | 0.80 (0.62-1.04) | 0.78 (0.62-0.99) | 0.83 (0.63-1.11) | 0.79 (0.63-1.01) |
| **Start year** | 1.06 (1.02-1.11) | 1.06 (1.02-1.11) | 1.07 (1.03-1.11) | 1.09 (1.04-1.14) | 1.03 (1.00-1.07) |
| **Nephrologist frequency (vs none)** |  |  |  |  |  |
| 1-2 times a month | 1.30 (0.92-1.85) | 1.33 (0.92-1.94) | 1.33 (0.94-1.87) | 0.91 (0.54-1.52) | 1.38 (0.97-1.95) |
| 1-3 times a week | 1.07 (0.74-1.55) | 1.08 (0.73-1.60) | 1.14 (0.80-1.64) | 0.80 (0.46-1.39) | 1.13 (0.78-1.62) |
| Daily | 1.00 (0.70-1.42) | 1.01 (0.70-1.48) | 1.03 (0.73-1.46) | 0.77 (0.45-1.31) | 1.08 (0.76-1.53) |
| **No∙ of beds (vs 1-5)** |  |  |  |  |  |
| 6-11 | 0.84 (0.65-1.07) | 0.84 (0.64-1.09) | 0.84 (0.66-1.07) | 0.95 (0.71-1.28) | 0.89 (0.70-1.14) |
| ≥12 | 0.80 (0.58-1.11) | 0.83 (0.59-1.16) | 0.79 (0.58-1.08) | 0.88 (0.61-1.28) | 0.96 (0.70-1.31) |
| **No∙ of staff (vs 1-4)** |  |  |  |  |  |
| 5-8 | 0.92 (0.73-1.16) | 0.93 (0.73-1.20) | 0.92 (0.74-1.16) | 0.78 (0.59-1.03) | 0.94 (0.74-1.20) |
| ≥9 | 0.91 (0.69-1.21) | 0.90 (0.67-1.22) | 0.92 (0.70-1.21) | 0.77 (0.56-1.07) | 0.88 (0.66-1.18) |
| **No∙ of active patients (vs 2-28)** |  |  |  |  |  |
| 29-66 | 0.89 (0.72-1.09) | 0.87 (0.69-1.09) | 0.88 (0.72-1.08) | 0.96 (0.73-1.25) | 0.91 (0.71-1.16) |
| ≥67 | 1.01 (0.78-1.32) | 1.00 (0.75-1.33) | 1.01 (0.78-1.30) | 1.08 (0.79-1.49) | 1.01 (0.76-1.33) |


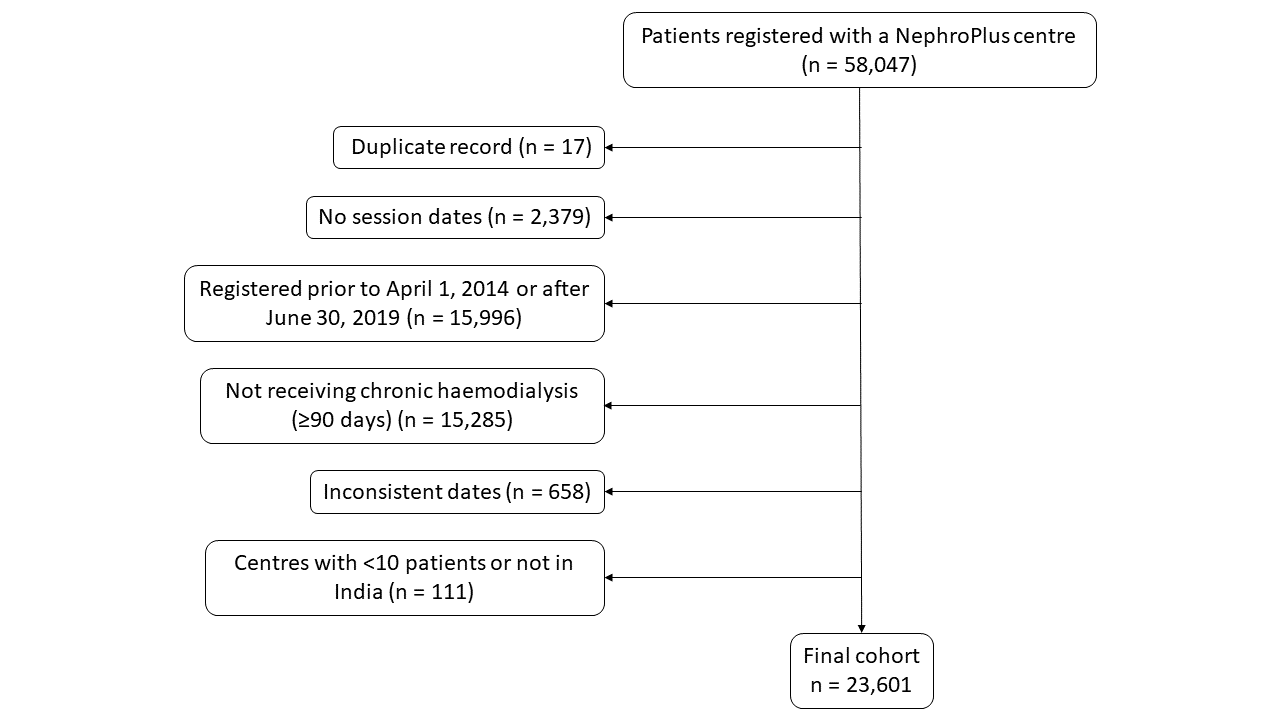


**Supplementary Figure 1** Participant flow chart

**

**

**Supplementary Figure 2** Adjusted analyses with only centre-level characteristics included.

**

**

**Supplementary Figure 3** Adjusted analyses with only patient-level characteristics included.
